# Policaptil Gel Retard Intake Reduces Postprandial Triglycerides, Ghrelin and Appetite in Obese Children: A Clinical Trial

**DOI:** 10.3390/nu12010214

**Published:** 2020-01-14

**Authors:** Elena Fornari, Anita Morandi, Claudia Piona, Mara Tommasi, Massimiliano Corradi, Claudio Maffeis

**Affiliations:** Pediatric Diabetes and Metabolic Disorders Unit, University City Hospital of Verona, 37126 Verona, Italy; anita.morandi@univr.it (A.M.); claudia.piona@univr.it (C.P.); mara.tommasi@aovr.veneto.it (M.T.); massimiliano.corradi@univr.it (M.C.); claudio.maffeis@univr.it (C.M.)

**Keywords:** obesity, children, postprandial phase, fibers, triglycerides, ghrelin, appetite

## Abstract

The aim of this study is to test the hypothesis that the intake of Policaptil Gel Retard^®^ (PGR) is able to affect appetite, metabolic and hormonal postprandial profile in obese children. 46 obese children were randomly assigned to treatment with PGR or placebo, in a double blind clinical trial. Two PGR tablets or placebo were given in fasting condition, before the ingestion of a mixed meal (15 kcal/kg lean body mass). Blood samples were taken at baseline and for 4 h, for measuring blood lipids, glucose, insulin, ghrelin, and glucagon like peptide-1 (GLP-1). Appetite was quantified using a visual analog scale. Children assuming PGR had a significantly lower increase of postprandial triglycerides (area under the curve (AUC): 3021 (2879) vs. 5038 (3738) mg × 240 min/Dl) and appetite (−234 (274) vs. 36 (329)) than children assuming placebo. The AUC of ghrelin was significantly lower after PGR ingestion, than after placebo (−8179 (8073) vs. −2800 (7579) pg × 240 min/mL). Blood glucose, insulin, non-esterified fatty acids (NEFA) and GLP-1 profiles were not significantly different in the two groups. In conclusion, a single intake of two tablets of PGR was associated with a significant reduction of appetite, ghrelin, and triglycerides in the postprandial period in obese children. Further investigation will assess if a chronic intake of PGR may affect body weight and glucose metabolism.

## 1. Introduction

Most of our daily hours are spent in the postprandial phase, from breakfast to midnight. The postprandial phase has a fundamental role in metabolic regulation [1]. In particular, exposition to high levels of substrates, i.e., glucose and fatty acids, after food intake increases the cardiovascular risk (CVR) as well as through inflammatory mechanisms and oxidative stress [1]. Accordingly, the reduction of the postprandial exposition to high levels of glucose and lipids limits the metabolic and vascular damage and the CVR of the individual [2,3].

The pre-meal intake of polysaccharides macromolecules produces an endoluminal gel that reduces and delays the absorption of nutrients by the gut [4,5]. Previous studies reported that the intake of soluble fiber is associated with a better postprandial glycaemic and lipid profile [6].

Moreover, fiber ingestion contributes to reduce appetite in both obese and non-obese subjects, delaying the colonic transit time of digested food, stimulating satiety hormone and endogenous glucagon like peptide-1 (GLP-1) secretion and inducing prolonged perception of post-meal satiation and satiety effects [7,8].

Policaptil Gel Retard^®^ (PGR) is a medical device in tablets, composed of polysaccharidic macromolecules (cellulose, hemicellulose, pectin, mucilages) and derived from raw materials (glucomannan, cellulose, Opuntia pulp stem, chicory root and freeze-dried mallow root, flaxseed and linden flower mucilage) rich in fibers.

The aim of this study was to verify whether the intake of PGR 20 min before meal ingestion, in comparison with placebo, was able to reduce postprandial lipid and glucose profile as well as ghrelin, insulin and appetite in a group of obese children.

## 2. Materials and Methods

### 2.1. Subjects

The study included 46 obese children, consecutively recruited from the Pediatric Diabetes and Metabolic Disorders Unit of the University Hospital of Verona. Inclusion criteria were age (8–12 years), ethnicity (white), obesity (BMI > cut-off of BMI for age and gender indicative of obesity, defined by World Health Organization reference) [9], acceptance to take part in the study. Exclusion criteria were birth defects, puberty, genetic disorders, chronic diseases other than obesity or chronic use of drugs, obesity secondary to endocrine or genetic diseases. None of the children was dieting at the time of the study.

### 2.2. Physical Characteristics

Anthropometry (weight, height, waist circumference), stage of pubertal development and blood pressure were measured in all the subjects. BMI was calculated as weight (in kilograms) divided by height (in meters) squared. BMI values were standardized (BMI z-scores) using age and sex-specific median, standard deviation, and power of the Box-Cox transformation (least mean square method) [9]. Body fat mass and body fat-free mass (FFM) were estimated by the bioelectrical impedance analysis scale (Bioelectrical Impedance, BIA: Tanita BC 420 MA, Tanita Corporation, Tokyo, Japan).

### 2.3. Experimental Protocol

The study protocol was randomized, double blind, placebo-controlled clinical trial. Each child arrived at the pediatric unit at 08:00, after an overnight fast. A Teflon catheter was inserted in the antecubital vein of one arm and a fasting blood sample was taken. Then subjects were randomly assigned to the arm with placebo or PGR and received the two tablets. PGR tablets and placebo tablets were identical in weight, texture, taste, and appearance but placebo tablets do not contain functional ingredients. After 20 min, a mixed meal was offered, to be consumed within 20 min. Blood samples were taken at 30 min intervals for the first two hours and at 60 min intervals for the following two hours, for the determination of metabolites and hormones for a total of 4 h. The Institutional Ethics Committee of Verona (Italy) has approved the study. The study was registered on ClinicalTrials.gov (ID: NCT02148614).

### 2.4. Test Meal

The energy content of the mixed meal was tailored to each subject, in order to standardize the caloric intake according to the children’s metabolically active mass, i.e., FFM. An intake of 15 kcal per kg of FFM was calculated. The amount of the different foods was proportionally increased or decreased for reaching the desired energy intake, maintaining a constant nutrient composition (protein 12%, lipid 35% and carbohydrate 53%).

### 2.5. Biochemical Analysis

Blood samples were collected in ethylenediamine tetraacetic acid (EDTA) (plus Pefabloc SC and dipeptidyl peptidase IV inhibitor in the tubes for hormones) at time 0’, 30’, 60’, 90’, 120’, 180’, 240’, centrifuged shortly after collection at 1600 g for 10 min at 4 °C and the plasma stored at −80 °C. Plasma glucose, triglycerides and non-esterified fatty acids (NEFA), collected at time 0’, 30’, 60’, 90’, 120’, 180’, 240’, were analyzed by using enzymatic-colorimetric methods (Sclavo Diagnostics International, Siena, Italy; Wako Chemicals GmbH, Neuss, Germany). Plasma insulin level was measured by commercial ELISA kit (Mercodia AB, Uppsala, Sweden). Ghrelin and total GLP-1, collected at time 0’, 30’, 60’, 120’, 180’, 240’, were analyzed by means of Milliplex MAP Kit Assay HMHEMAG-34K (Merck Millipore, Darmstadt, Germany).

### 2.6. Subjective Appetite

At baseline and at 30 min intervals for the first two hours and 60 min intervals for the following two hours in the postprandial phase, the level of hunger was assessed by a visual analog scale simplified for the use in children [10]. The scale was made up of five numbered boxes with different colors. Each box corresponded to a different value of perceived hunger, starting with 1 (most negative rating) and increasing to 5 (most positive rating).

### 2.7. Statistical Analysis

We recruited a convenience sample size of 46 subjects. This sample was 85% powered to detect a 0.9 standard deviation difference and 50% powerful to detect a 0.6 standard deviation difference between the two groups, in all normal continuous variables, given a 5% α error. We performed the a posteriori sensitivity analysis required to assess our sample power by G-power software (http://gpower.hhu.de/). We established that all continuous variables were normally distributed by Kolgomorov–Smirnov test. We compared the two groups as regards physical, biochemical and metabolic variables and incremental area under the curves (iAUCs) of metabolites and hormones by Student’s t test. Comparison between proportions were done by Chi squared test. The SPSS 21.0 (SPSS, Inc., Chicago, IL, USA) software was used to perform the analysis. The level of significance of the tests was set at *p* < 0.05.

## 3. Results

### 3.1. Physical Characteristics

The physical characteristics of the subjects are shown in Table 1. Age, gender distribution, weight, height, BMI, BMI z-score, waist circumference, waist to height ratio, fat mass and fat-free mass were not significantly different between the two groups.

### 3.2. Dietary Intake

Energy and macronutrient composition of the test meal are reported in Table 1. Energy and nutrient intakes of the two groups were not significantly different.

### 3.3. Postprandial Biochemical and Hormonal Parameters

**Figure 1:**
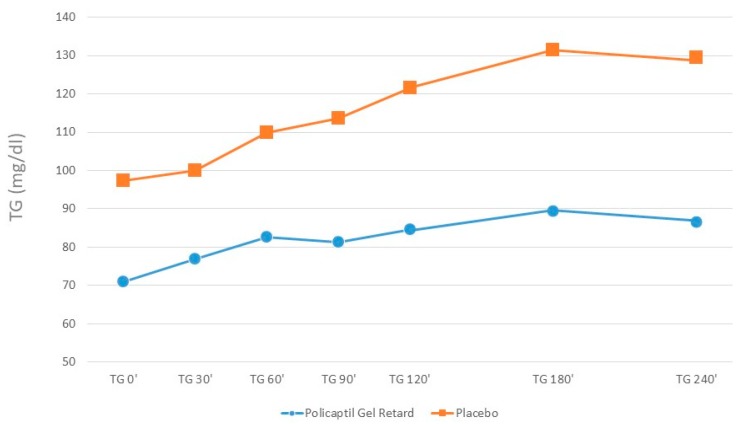
Triglycerides serum concentration before and after the ingestion of PGR or placebo.

The iAUCs of glucose, insulin, NEFA, GLP1, triglycerides and Ghrelin are shown in Table 2. Food intake induced an increase in glucose concentration, not significantly different in the two groups, and an increase in triglyceride concentrations, significantly (*p* < 0.05) lower after the pre-prandial intake of PGR than after placebo (Figure 1).

**Figure 2:**
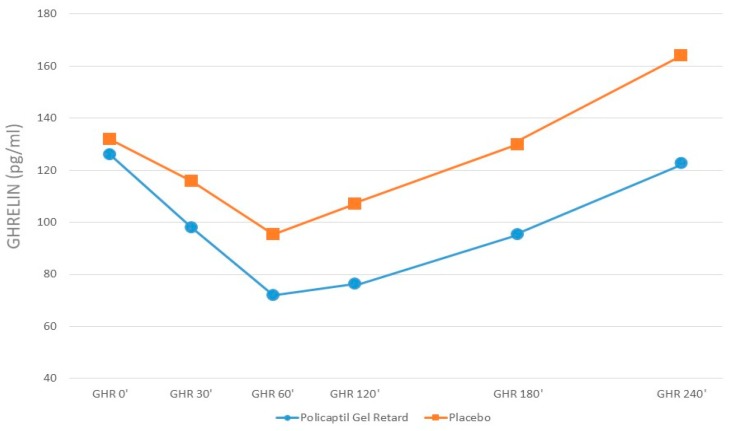
Ghrelin serum concentration before and after the ingestion of PGR or placebo.

Plasma NEFA decreased after meal intake but no significant difference was found between the two groups. Ghrelin concentrations decreased after meal intake in both groups. The iAUC of Ghrelin was significantly lower after PGR ingestion than after placebo (Figure 2). GLP-1 and insulin concentrations increased after meal intake but the iAUCs were not significantly different in the two groups.

### 3.4. Appetite

**Figure 3:**
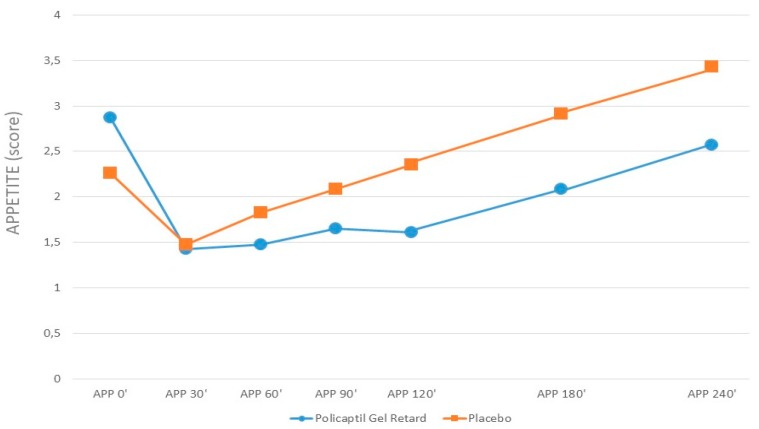
Appetite visual analog scale scores before and after the ingestion of PGR or placebo.

The iAUCs of appetite scores are shown in Table 2. The appetite score was not significantly different in the two groups in fasting conditions. The iAUC of appetite was significantly lower after PGR ingestion than after placebo (Figure 3).

## 4. Discussion

The main result of this study was the efficacy of the intake of PGR 20 min before meal in inducing a lower increase in triglycerides levels and a significant reduction of appetite, compared to placebo, in the 240 min postprandial phase.

The exposition to cardiovascular risk factors in childhood and adolescence is associated with morbidity and mortality later in life [11,12]. Circulating lipids are one of the most important of these factors [2,3,13,14]. Recent genetic studies have demonstrated that high plasma levels of non-fasting triglycerides are causally associated with an increased risk of cardiovascular disease (CVD) [15]. Accordingly, the American Heart Association highlights the pivotal role of triglycerides in increasing CVD risk and indicates their reduction as one of the targets of CVR prevention [13,14].

An increase of TG level lasting several hours follows meal intake [16]. Therefore, due to the several eating episodes during the day, humans are normally exposed to levels of TG higher than those measured in fasting conditions for most of the day, i.e., from breakfast to late in the night [17]. Moreover, the amount of fat intake of the meal is associated with the postprandial TG levels, i.e., increasing fat ingested increases the TG postprandial levels [17]. Therefore, especially in obese individuals, a reduction of the TG level in postprandial conditions, influencing inflammatory mechanisms and limiting the oxidative stress, is theoretically useful for reducing the metabolic risk [2,13].

One of the potential strategies for promoting a reduction of TG postprandial profile is to add fibers to the meal. In fact, the beneficial effect of high dietary fiber intake on lowering plasma lipid concentrations is well known [5]. In this study, we found that PGR, ingested 20 min before a mixed meal intake, was able to reduce significantly the TG increase in the post-prandial phase in comparison to placebo. Previous studies on adults evaluated postprandial lipaemia after a higher amount of fats [18,19]. However, we decided to use a test meal, which respected the national RDI, with a fat intake of 35% of total energy [20] and closer to that reported in a previous study conducted in obese children leaving in the same geographical area [21]. This choice allowed exploring the response to PGR in condition closer to real life. The effect of the pre-meal ingestion of the PGR may be still higher after a high fat meal.

In our study, PGR pre-meal ingestion caused a significantly higher reduction of ghrelin levels and a lower increase in GLP-1 in postprandial phase, compared to placebo. Accordingly, subjective postprandial perception of appetite, measured by the visual analog scale, was significantly lower when PGR ingestion preceded the meal, compared to placebo.

The effect of fibers intake on appetite is still debated. A recent review, which analyses the influence of dietary fibers on appetite and energy intake, concludes that data reported in human intervention trials are still too inconsistent to allow generalized conclusions on their effect [22]. Nevertheless, several mechanisms are described, by which dietary fiber is able to modulate satiety in pre- and post-absorbing phase [4]. The reduction of palatability, the deceleration of gastric emptying rate and the stimulus to the production of hormones which are involved in appetite regulation, are only a part of them [4,8].

Our results confirm the post-prandial effect of fiber ingestion on satiety and on reducing hormones that stimulate appetite, corroborating data on adults [4]. In addition, improving satiety, fiber ingestion could determine a lower caloric intake at the next meal, and in the long term, it could be effective also in reducing excessive weight.

No significant differences have been detected between the two groups in glucose and insulin postprandial profiles. The hypothesis that the polysaccharidic macromolecules gelation can create an intestinal film, which slow glucose absorption and consequently reduce pancreatic insulin release [23], is not confirmed by our data. Two previous studies conducted by Stagi et al. demonstrated that PGR could be useful to potentiate weight loss and to improve glucose metabolism. After one year of follow-up, administration of PGR in association with a low-glycemic-index diet, reduced weight gain and ameliorated the metabolic syndrome and insulin-resistance parameters in obese children and adolescents with family history of obesity and T2DM [24]. In addition, Stagi reported that 71 obese children and adolescents treated for one year with PGR added to metformin showed a significantly larger reduction in body mass index standard deviation score (BMI-SDS) and glucose metabolism improvement compared with peers treated with metformin (*n* = 58) or diet alone (*n* = 51) [25]. There is more than one potential reason why we did not find significant PGR effects on glucose and insulin postprandial profiles. First, a single administration may not be sufficient in children to modify significantly glucose absorption and insulin secretion after taking a mixed meal. Second, the mixed meal administered in our study provides a lower amount of glucose and has a lower glycemic index compared to the glucose load provided in the OGTT performed in the studies by Stagi and co-authors. Third, the improvement of glucose metabolism highlighted by Stagi’s studies may be due to the PGR effect on the amelioration of anthropometric parameters over the treatment period and not to a direct PGR effect on glucose metabolism.

Potential limitations of this study are: (i) ethnicity: only Caucasian children have been recruited. We should be cautious to generalize these results to children of other ethnic groups; (ii) the lack of the comparison between the hormonal postprandial profiles and the reported appetite scores with gastric emptying; (iii) the sample size, despite the a posteriori sensitivity analysis suggested that it was adequate for the purposes of this study.

The strengths of this study were: (i) the study design: randomized, double blind, placebo-controlled clinical trial; (ii) the novelty of the study conducted in prepubertal children without comorbidities; and (iii) the potential clinical implications in the short-term: limiting postprandial hypertriglyceridemia could be effective in treatment of obesity and comorbidities.

Despite the influence of dietary fibers on appetite and energy intake needs for more investigations, the positive health effects of fiber ingestion in reducing cardiovascular risk are well established in the scientific literature [7,26]. Considering that most of our daily hours are spent in the postprandial phase, it may be useful to find strategies to reduce the CVR factors, which emerge in this part of the day, particularly in obese children, who generally introduce less quantity of fibers compared to non-obese children and are intrinsically exposed to higher CVR [10,11,27].

## 5. Conclusions

Taking advantage of pre-meal ingestion of fibers in order to reduce triglycerides increase and to delay appetite in the postprandial phase could be a useful strategy to limit the exposition to cardiovascular risk factors particularly in obese children. The hypothetical long-term effect could lead to further benefit including weight reduction.

## Figures and Tables

**Table 1:** Physical characteristics of the subjects and energy and macronutrient composition of the test meal.

	PGR (*n* = 23)	Placebo (*n* = 23)	*p*
Physical Characteristics			
Age (years)	10.4 (1.1)	9.8 (1.1)	ns
Gender (M/F)	12/11	10/13	ns
Weight (kg)	56.1 (12.1)	55.5 (14.5)	ns
Height (cm)	147.4 (10.3)	142.5 (10.0)	ns
BMI (kg × (m^2^)^−1^)	25.6 (2.8)	26.8 (4.0)	ns
BMI z-score	1.68 (0.49)	1.89 (0.53)	ns
WC (cm)	83.5 (7.6)	81.2 (11.7)	ns
WHtR	0.57 (0.04)	0.57 (0.06)	ns
Fat Mass (kg)	19.1 (5.8)	21.5 (8.1)	ns
Fat Mass (%)	33.8 (5.9)	37.8 (6.3)	ns
Fat free mass (kg)	37.0 (7.9)	33.9 (7.1)	ns
**Energy and Macronutrient Composition of the Test Meal**			
Total Energy (kcal)	555.5 (119.3)	509.0 (107.3)	ns
Carbohydrates (g)	73.4 (17.1)	67.3 (14.4)	ns
Carbohydrates (kcal)	294.4 (63.2)	269.7 (56.9)	ns
Carbohydrates (%)	53.0 (11.4)	53.0 (11.2)	ns
Protein (g)	16.6 (3.9)	15.2 (3.2)	ns
Protein (kcal)	66.7 (14.3)	61.1 (12.9)	ns
Protein (%)	12.0 (2.5)	12.0 (2.5)	ns
Fat (g)	21.6 (5.0)	19.8 (4.2)	ns
Fat (kcal)	194.4 (41.7)	178.1 (37.6)	ns
Fat (%)	35.0 (7.5)	35.0 (7.4)	ns

Data shown as mean and standard deviation in brackets. Abbreviations: PGR, Policaptil Gel Retard; BMI, body mass index; WC, waist circumference; WHtR, waist-to-height ratio.

**Table 2:** iAUCs of glucose, insulin, NEFA, GLP1, triglycerides, Ghrelin and appetite.

iAUC	PGR (*n* = 23)	Placebo (*n* = 23)	*p*
Glucose (mg × 240 min/dL)	3576 (2241)	3022 (1524)	ns
Insulin (mU × 240 min/L)	12,664 (15,047)	13,366 (8351)	ns
NEFA (mmol × 240 min/L)	−67 (39)	−82 (37)	ns
GLP1 (pg × 240 min/mL)	−228 (4935)	2586 (4418)	ns
Triglycerides (mg × 240 min/dL)	3021 (2879)	5038 (3738)	0.046
Ghrelin (pg × 240 min/mL)	−8179 (8073)	−2800 (7579)	0.026
Appetite (score)	−234 (274)	36 (329)	0.004

Data shown as mean and standard deviation in brackets. Abbreviations: iAUCs, incremental area under the curves; PGR, Policaptil Gel Retard; NEFA: non esterified fatty acids; GLP1: Glucagon-Like Peptide 1.

## Supplementary Materials

Supplementary File 1
