# Supplementary figures and images for: Policaptil Gel Retard Intake Reduces Postprandial Triglycerides, Ghrelin and Appetite in Obese Children: A Clinical Trial
Source: Nutrients. 2020 Jan 14;12(1):214. doi: 10.3390/nu12010214 (PMC7019363; doi:10.3390/nu12010214)

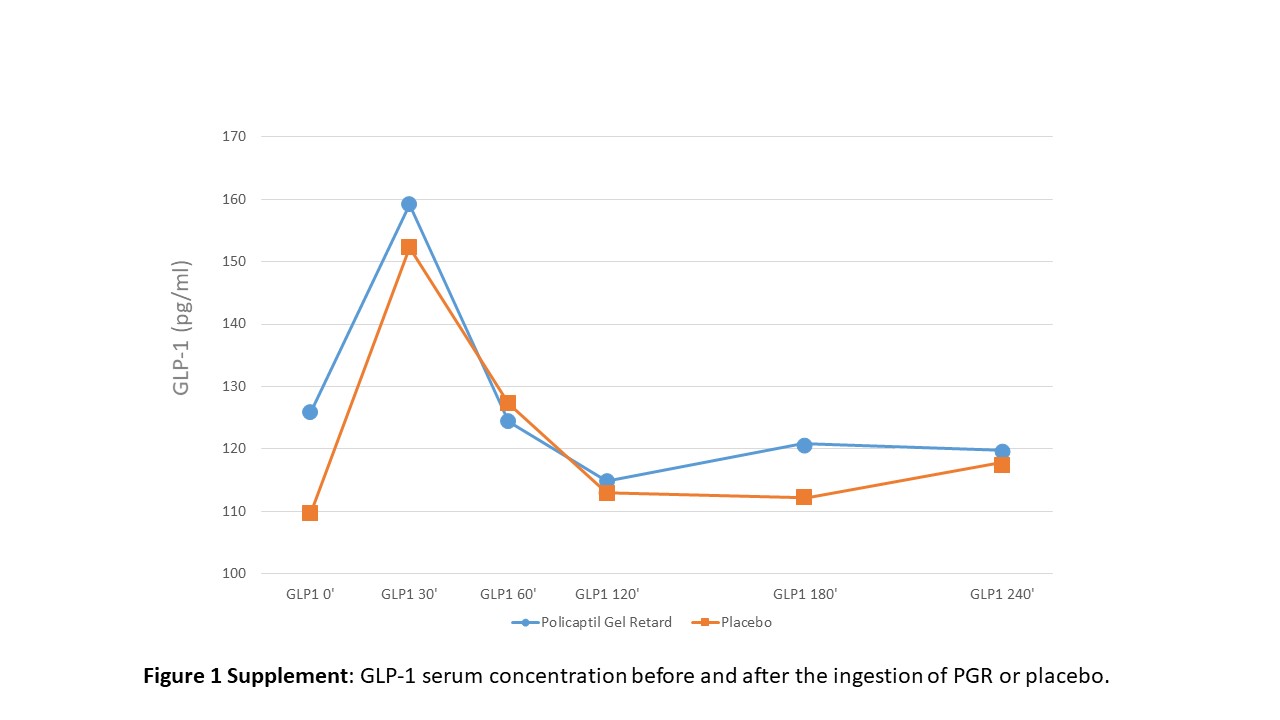

Supplement: Supplementary File 1 [file nutrients-12-00214-s001.zip › nutrients-672215-SI.jpg]
